# Molecular Epidemiology and Antibiotic Susceptibility of *Vibrio cholerae* Associated with a Large Cholera Outbreak in Ghana in 2014

**DOI:** 10.1371/journal.pntd.0004751

**Published:** 2016-05-27

**Authors:** Daniel Eibach, Silvia Herrera-León, Horacio Gil, Benedikt Hogan, Lutz Ehlkes, Michael Adjabeng, Benno Kreuels, Michael Nagel, David Opare, Julius N Fobil, Jürgen May

**Affiliations:** 1 Bernhard Nocht Institute for Tropical Medicine (BNITM), Hamburg, Germany; 2 National Center of Microbiology, Institute of Health Carlos III, Madrid, Spain; 3 European Public Health Microbiology Training Programme (EUPHEM), European Centre for Disease Prevention and Control (ECDC), Stockholm, Sweden; 4 German Center for Infection Research (DZIF), partner site Hamburg-Borstel-Lübeck, Germany; 5 Ghana Health Service, Disease Surveillance Service, Accra, Ghana; 6 University Medical Centre Hamburg-Eppendorf (UKE), Hamburg, Germany; 7 Kumasi Centre for Collaborative Research in Tropical Medicine (KCCR), Kumasi, Ghana; 8 Ghana Health Service, National Public Health and Reference Laboratory (NPHRL), Accra, Ghana; 9 Department of Biological, Environmental and Occupational Health Sciences, School of Public Health, University of Ghana, Accra, Ghana; Massachusetts General Hospital, UNITED STATES

## Abstract

**Background:**

Ghana is affected by regular cholera epidemics and an annual average of 3,066 cases since 2000. In 2014, Ghana experienced one of its largest cholera outbreaks within a decade with more than 20,000 notified infections. In order to attribute this rise in cases to a newly emerging strain or to multiple simultaneous outbreaks involving multi-clonal strains, outbreak isolates were characterized, subtyped and compared to previous epidemics in 2011 and 2012.

**Methodology/Principal Findings:**

Serotypes, biotypes, antibiotic susceptibilities were determined for 92 *Vibrio cholerae* isolates collected in 2011, 2012 and 2014 from Southern Ghana. For a subgroup of 45 isolates pulsed-field gel electrophoresis, multilocus sequence typing and multilocus-variable tandem repeat analysis (MLVA) were performed. Eighty-nine isolates (97%) were identified as *ctx*B (classical type) positive *V*. *cholerae* O1 biotype El Tor and three (3%) isolates were cholera toxin negative non-O1/non-O139 *V*. *cholerae*. Among the selected isolates only sulfamethoxazole/trimethoprim resistance was detectable in 2011, while 95% of all 2014 isolates showed resistance towards sulfamethoxazole/trimethoprim, ampicillin and reduced susceptibility to ciprofloxacin. MLVA achieved the highest subtype discrimination, revealing 22 genotypes with one major outbreak cluster in each of the three outbreak years. Apart from those clusters genetically distant genotypes circulate during each annual epidemic.

**Conclusions/Significance:**

This analysis suggests different endemic reservoirs of *V*. *cholerae* in Ghana with distinct annual outbreak clusters accompanied by the occurrence of genetically distant genotypes. Preventive measures for cholera transmission should focus on aquatic reservoirs. Rapidly emerging multidrug resistance must be monitored closely.

## Author Summary

The bacterium *Vibrio cholerae* is mainly transmitted faecal-orally via human-to-human contact or via environmental water sources in which *V*. *cholerae* is able to persist. West Africa, including Ghana, is regularly affected by Cholera epidemics, in particular during rainy seasons. In 2014, Ghana experienced an exceptionally large outbreak with over 20,000 cases, which raised questions about newly emerging *V*. *cholerae* strains in this region. In this study, we described the duration, the geographical spread and demographics of the outbreak using data from the National Ghanaian Surveillance system. Further, we characterized outbreak isolates from the outbreak years 2011, 2012 and 2014 by three different subtyping methods. These analyses revealed strains with different genetic background and increasing antibiotic resistance circulating during each outbreak year. These data suggest that *V*. *cholerae* has an endemic reservoir in the environment and selection pressure results in a highly heterogeneous population of *V*. *cholerae* with a few strains evolving into pathogenic clones during each outbreak period. Public health authorities must be vigilant to prevent cholera transmission through aquatic reservoirs, particularly within urban agglomerations during the start of the rainy season. The rapidly emerging antibiotic resistance has to be monitored closely.

## Introduction

The World Health Organization (WHO) estimates that 3–5 million annual cases of cholera occur worldwide, resulting in 100,000–120,000 deaths [1]. Since its introduction to Africa during the current seventh cholera pandemic in 1970, *Vibrio cholerae* caused regular vast epidemics across the continent with cumulative 3,762,902 case-notifications to WHO by 2013 [2]. In 2013 alone, 22 African countries reported 56,329 cholera cases. However, these numbers are considered to be substantially underestimated due to poorly functioning national epidemiological and laboratory surveillance systems, which are not able to detect the majority of mild disease presentations. Furthermore, systematic underreporting is common to avoid economic and political damage [3,4].

The first cholera case has been reported from Ghana in 1970 [5]. With 90% of the population at risk for cholera transmission, Ghana always ranks among the most affected countries on the African continent [4]. Ghana notified an annual average of 3,066 (range: 50–10,628) cholera cases between 2000 and 2013 with an overall case fatality rate of 1.7% [2]. In the year 2014, Ghana experienced an exceptionally large cholera outbreak with 28,975 infections notified to the World Health Organization between June and November [6].

Causes of this sudden increase in case numbers might be very diverse and calls for investigation in a timely manner so as to implement preventive measures accordingly. This recent epidemic might be explained by the extrinsic introduction of a new *V*. *cholerae* strain into a susceptible population, as seen for cholera outbreaks in Zimbabwe and Haiti in the years 2009 and 2011 respectively [7,8]. Alternatively, multiple, endemic, genetically non-related strains might be responsible for concomitant epidemics, as reported from Kenya [9]. In the latter scenario *V*. *cholerae* might persist in aquatic reservoirs and regular epidemics are triggered by low hygienic conditions and climatic factors such as increased rainfall or flooding [10–12]. In recent years, a number of molecular typing tools have been developed to elucidate the source and evolution of *V*. *cholerae* outbreaks [13].

This study aims to describe the 2014 cholera epidemic in Ghana and uses molecular subtyping techniques to detect responsible newly emerging or multi-clonal strains, which are then compared to strains that circulated during the 2011 and 2012 epidemics. Results will advise public health authorities whether to focus on monitoring of endemic environmental reservoirs or rather on surveillance of mobile populations, with cross-border epidemiological collaborations to prevent importation of *V*. *cholerae*.

## Methods

### Epidemiological description of the 2014 outbreak

Within the Ghana Health Service, the Disease Surveillance Service supported by the National Public Health & Reference Laboratory (NPHRL) conducts cholera surveillance in Ghana. A case of cholera is defined according to the WHO standard case definition: If cholera is not known to be present in the area, a case of cholera is considered in a patient ≥5 years with severe dehydration or death from acute watery diarrhea, while during a cholera epidemic every patient aged ≥5 years with acute watery diarrhea and/or vomiting is considered as a case.

Standardized line lists with suspected cholera cases are provided on a weekly basis by the District Health Management Teams to the Central Disease Surveillance Service in Accra, which collates information on name, place of residence, sex, age, disease onset, disease outcome and hospitalization of cases. A small subset of specimens is usually tested in peripheral laboratories by various methods and results are notified in the line lists. For laboratory confirmation, district and regional laboratories are encouraged to send suspected cholera stool samples to the NPHRL, where culture and serotype identification is performed.

For the purposes of this study, all suspected cholera cases in the surveillance database from six Southern Ghanaian regions (Greater Accra, Central, Western, Eastern, Ashanti, Volta) from the year 2014 were extracted and described by sex, age and place of residence. Continuous variables were summarized as means with standard deviation (median with interquartile range for non-normally distributed variables) and dichotomous or categorical variables were summarized as proportions/percentages. Missing values were excluded from the analysis, thus the denominators for some comparisons differ.

Surveillance data were computerized using Excel (Microsoft, USA) and statistical analysis was performed with Stata v. 12.1 (Statacorp, Texas, USA). For spatio-temporal visualisation case data were imported into Arc GIS 10.0 (ESRI: ArcGis Desktop: Release 10.2011). Suspected cases were linked to the respective notifying districts. For the spatio-temporal visualisation, case data/district were then aggregated in six temporal groups, each covering five outbreak weeks.

### Characterization of isolates and molecular epidemiology

#### Identification of *V*. *cholerae* isolates

All *V*. *cholerae* isolates, which were sent to the NPHRL from the Greater Accra-, Central-, Ashanti- and Volta regions, from the years 2011 (n = 18) and 2012 (n = 12), in addition to 62 randomly selected isolates from the year 2014 (out of 261 isolates), were chosen for further analysis. Samples were randomly selected by district and time, reflecting the spatial-temporal distribution within the outbreak. All 92 isolates were initially cultured on Thiosulfate-citrate-bile salts-sucrose (TCBS) agar and subcultured on Tryptic Soy Agar (TSA). DNA was extracted out of a suspension of *V*. *cholerae* cells, which had been incubated for 10 min at 100°C. After centrifugation, the supernatant, containing the DNA, was preserved at -20°C until further processed. *V*. *cholerae* isolates were confirmed by species-specific PCR and tested for the presence of cholera toxin (*ctx*AB) by PCR as described before [14,15]. For the year 2013 50 cholera cases have been reported from Ghana, but no isolates were send for confirmation to the NPHRL. For further analyses the isolates were transported to the national reference laboratory at the ISCIII in Madrid, Spain.

#### Phenotyping

The isolates were serotyped with polyvalent O1 antiserum and monovalent Inaba and Ogawa antisera (Denka Seiken, Tokyo, Japan). Biotypes were determined by (i) susceptibility to polymyxin B, (ii) haemolysis of sheep erythrocytes and (iii) the Voges-Proskauer test, which measured the production of acetylmethylcarbinol.

Antimicrobial susceptibility testing was performed with the Kirby-Bauer disk diffusion method on Mueller-Hinton agar. The *Escherichia coli* reference strain ATCC 25922 served as a control. Isolates were tested against 7 antimicrobial drugs as follows: ampicillin (10 μg), ciprofloxacin (5 μg), chloramphenicol (30 μg), gentamycin (30 μg), nalidixic acid (30 μg), sulfamethoxazole/trimethoprim (1.25 μg + 23.75 μg) and tetracycline (30 μg)(all Oxoid, Basingstoke, United Kingdom). Diameters were interpreted according to the 2015 Clinical and Laboratory Standards Institute (CLSI) guidelines as resistant, susceptible or intermediate (http://www.clsi.org). Intermediate isolates will be referred to as reduced susceptible in the following. When no interpretive criteria for *V*. *cholerae* were available, breakpoints for Enterobacteriaceae according to the 2015 European Committee on Antimicrobial Susceptibility Testing (EUCAST) guidelines were applied (http://www.eucast.org).

#### Genotyping

The type of the cholera toxin-encoding gene (*ctx*B) was determined by the mismatch amplification mutation PCR as described elsewhere [16]. From all isolates identified within a two-week period in a specific district, one isolate was randomly selected for further molecular characterization, resulting in a subset of 45 isolates. The geographical origin of these isolates is shown in the supplement (S1 Fig).

Pulse-field gel electrophoresis (PFGE) was conducted according to the PulseNet protocol for *V*. *cholerae*, using a single restriction enzyme digestion with *Not*I as described before [17]. PFGE banding patterns and dendrogram were analyzed with Bionumerics version 6.6 (Applied Math, Texas, USA). The definition of a PFGE cluster was based on a similarity cutoff of 95% (Dice coefficient, represented by UPGMA, 1.0% optimization and 1.5% tolerance). For each detected PFGE pulsotype, one isolate was chosen for Multilocus Sequence Typing (MLST), following the published protocol for 7 loci as previously described [18]. Newly identified alleles and sequence types were submitted to the public cholera database for assignment to new sequence types (http://pubmlst.org/vcholerae/).

Multilocus variable-number tandem-repeat (VNTR) analysis (MLVA) was carried out by amplification of 5 loci using primers and PCR conditions as described in previous studies [19]. The purified PCR products were sequenced in both directions and sequence data were analyzed using the DNASTAR Lasergene version 7.0 software. MLVA types were assigned by combining numbers of repeat units of each locus in the order VC0147, VC0436-7, VC1650, VCA0171, VCA0283. A minimum spanning tree was generated using Bionumerics version 6.6 (Applied Math, Texas, USA) based on the categorical coefficient. Clonal complexes were defined as isolates connected through a chain of single-locus variants.

## Results

### Epidemiology of the 2014 outbreak

**Fig 1:**
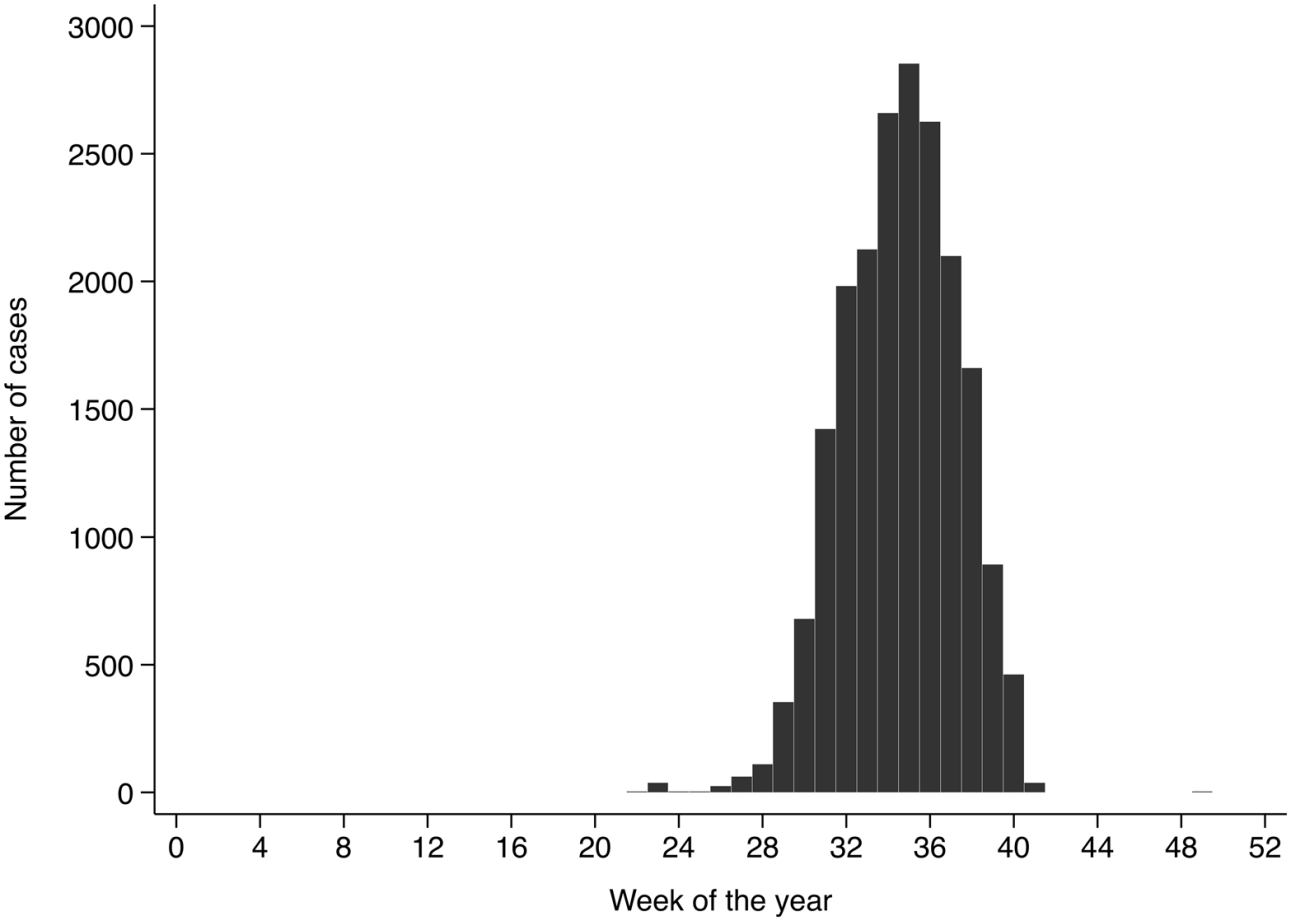
Weekly notification of suspected cholera cases. The Disease Surveillance Service of the Ghana Health Service reports 20,120 cholera cases according to the WHO case definition between May 2014 and December 2014 with a peak number of 2,853 cases in the 35^th^ calendar week (25–31 August).

A total of 20,185 cases of cholera were reported to the Ghanaian Disease Surveillance Center from the Ashanti-, Central-, Eastern-, Greater Accra-, Western- and Volta regions in 2014. The date of disease onset was reported for 20,120 cases, the remaining 65 cases were removed from the dataset for all other analyses. The earliest reported onset date was the 20^th^ of May 2014 and the latest was the 11^th^ of December 2014, with a peak number of 2,853 cases in the 35^th^ calendar week (25–31 August; Fig 1). Age was reported for 19,863 cases and distributed with a median age of 26 years and an interquartile range (IQR) of 20–35 years. The median age of cases did not change during the course of the outbreak. The majority of cases was male (58.4%; n = 11,796), and median age was not markedly different between males (26 years; IQR 20–35) and females (25 years; IQR 19–35). The case fatality rate (CFR) was 0.8% (165 deaths) with a higher median age among deceased of 34 years (IQR 24–47).

Laboratory testing was performed in regional laboratories with rapid diagnostic tests from different suppliers for 496 out of 20,120 (2.5%) suspected cases with a positivity rate of 53% (264/496).

**Fig 2:**
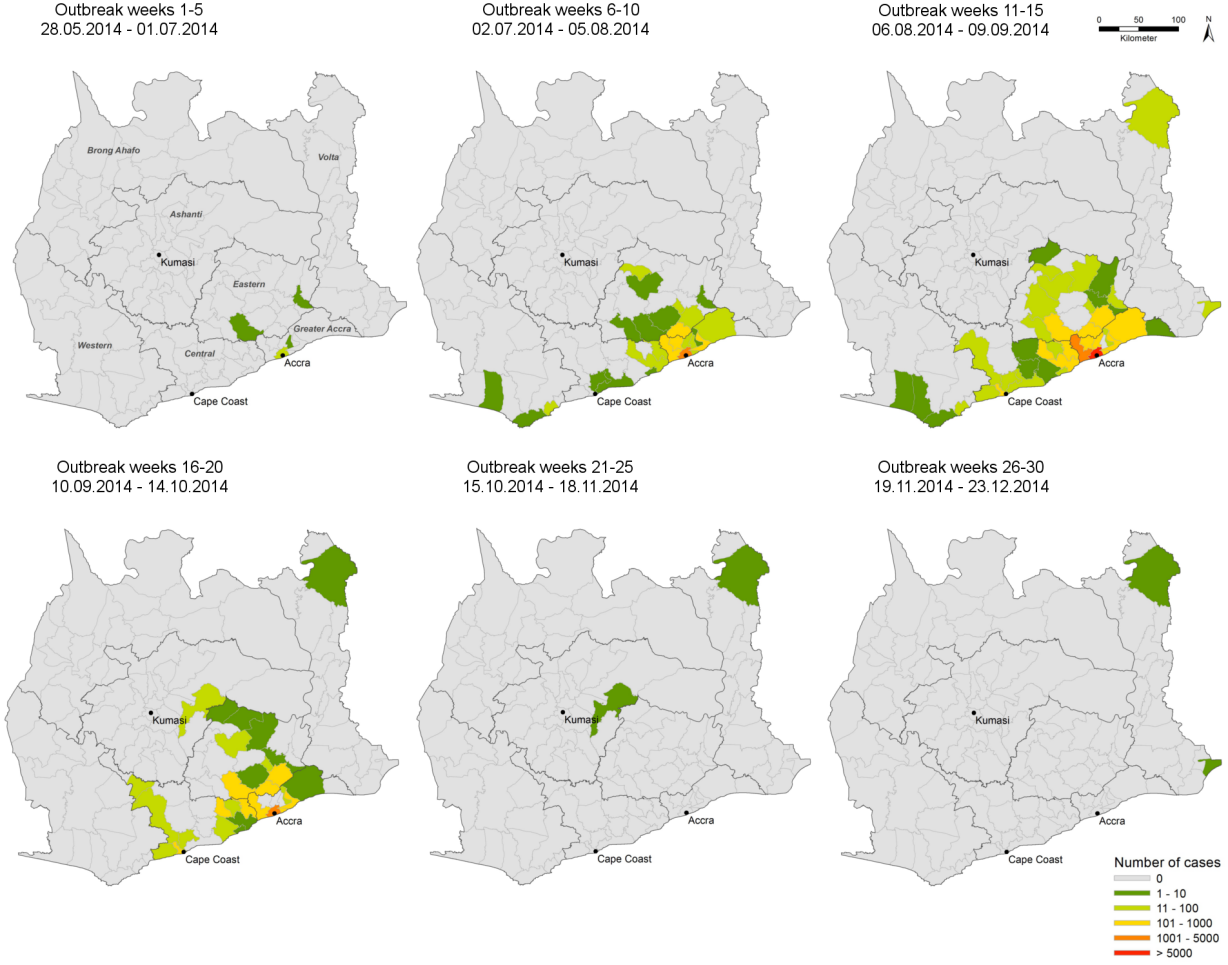
Spatial and temporal location of suspected cholera cases (Mai 2014-December 2014; n = 20,120). As notified to the Disease Surveillance Service of the Ghana Health Service according to the WHO case definition suspected cholera cases are plotted by district by 5-week period panels. The figure was produced with Arc GIS 10.0 (ESRI: ArcGis Desktop: Release 10.2011).

Spatio-temporal analysis traced back the first cases during the first five weeks of the outbreak to four districts in the Eastern and Greater Accra regions (Fig 2). During the peak of the outbreak (outbreak week 6–20) the majority of cases were centred around the city of Accra, spreading in northward-direction to the Ashanti region and to the East along the coast. During the last 10 weeks of the outbreak, only three districts within the Ashanti and the Volta region reported cases.

### Antimicrobial susceptibility

Antimicrobial susceptibility testing identified mono-resistance towards sulfamethoxazole/trimethoprim in 100% (18/18) of isolates from 2011 (Table 1). The resistance pattern changed in 2012 with 75% (9/12) of isolates expressing resistance against sulfamethoxazole/trimethoprim, nalidixic acid in combination to reduced susceptibility against ampicillin and ciprofloxacin. The same resistance profile was detected in 95% (59/62) of isolates in 2014. No resistance against chloramphenicol, gentamycin or tetracycline was observed for any of the isolates.

**Table 1:** Antimicrobial resistance for each antibiotic (A) and resistance profile (B) of *Vibrio cholerae* isolates, by year of disease onset (n = 92).

**A.**			
**Resistance for each antibiotic [n (%)]**			
**Antibiotic**	**2011 (n = 18)**	**2012 (n = 12)**	**2014 (n = 62)**
SxT	18 (100.0)	10 (83.3)	60 (96.8)
Nal	0 (0.0)	11 (91.7)	62 (100.0)
Cip	0 (0.0)	11 (91.7)	61 (98.4)
Amp	0 (0.0)	11 (91.7)	59 (95.2)
**B.**			
**Resistance profile [n (%)]**			
**Antibiotics**	**2011 (n = 18)**	**2012 (n = 12)**	**2014 (n = 62)**
SxT	18 (100.0)	1 (8.3)	0 (0.0)
Nal	0 (0.0)	0 (0.0)	1 (1.6)
Nal+Cip	0 (0.0)	0(0.0)	1 (1.6)
Nal+Cip+Amp	0 (0.0)	2 (16.7)	0 (0.0)
Nal+Cip+SxT	0 (0.0)	0 (0.0)	1 (1.6)
Nal+Cip+Amp+SxT	0 (0.0)	9 (75.0)	59 (95.2)

Amp, ampicillin; Cip, ciprofloxacin; Nal, nalidixic acid; SxT, sulfamethoxazole/trimethoprim; no antimicrobial resistance was found for chloramphenicol, gentamycin and tetracycline.

### Genotyping

The selected 92 *V*. *cholerae* isolates consisted of 88 (95.7%) *V*. *cholerae* O1 biotype El Tor serotype Ogawa, 1 (1.1%) *V*. *cholerae* O1 biotype EL Tor serotype Inaba and 3 (3.3%) non-01/non-O139 *V*. *cholerae*. Apart from the non-O1/non-O139 *V*. *cholerae* strains with no cholera toxin production, all isolates produced the classical type cholera toxin.

**Fig 3:**
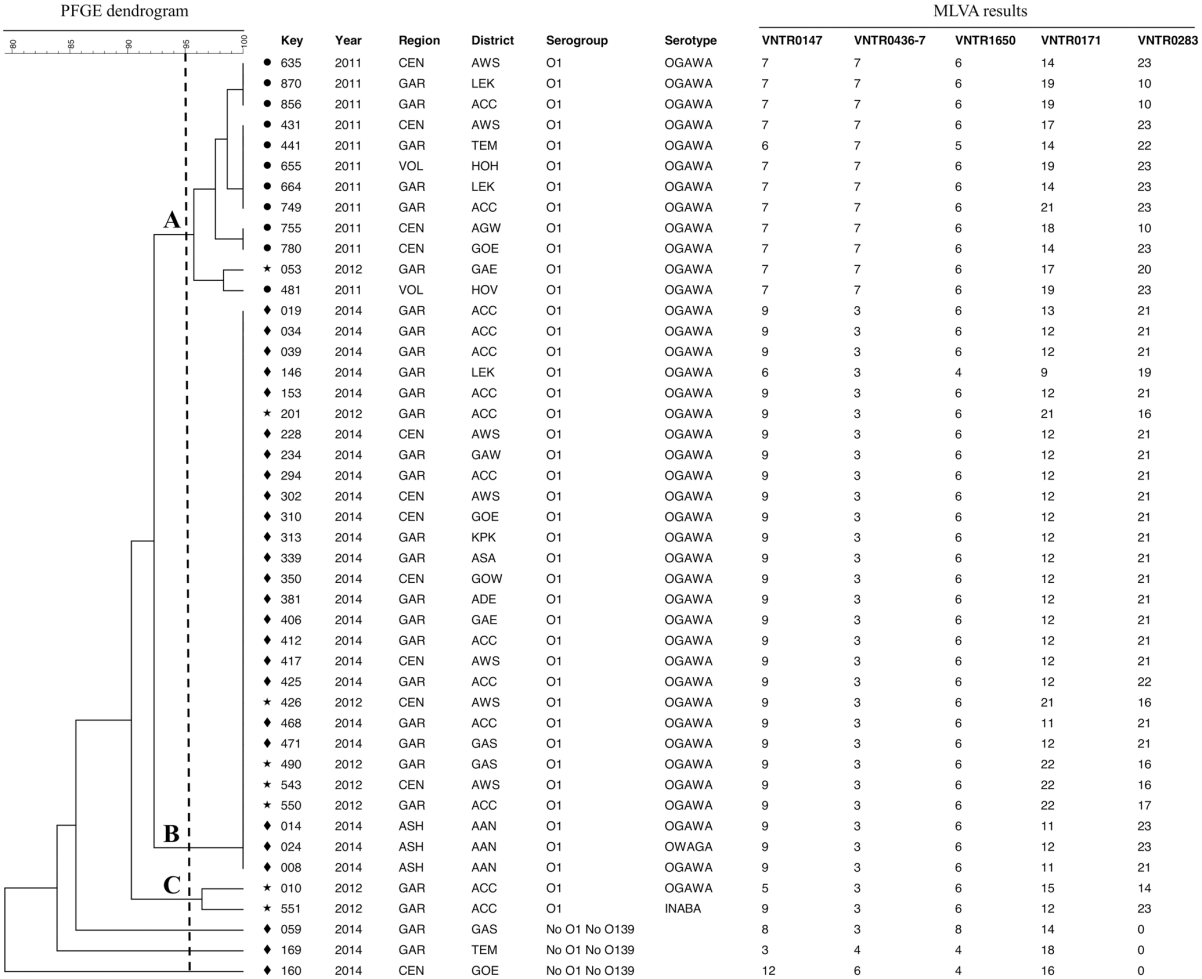
Pulse-field gel electrophoresis (PFGE) dendrogram for *Vibrio cholerae* isolates (n = 45). The three clusters A, B and C (bold letters) are based on a similarity cut-off of 95% (Dotted line; Dice coefficient, represented by UPGMA, 1.0% optimization and 1.5% tolerance). The geographical location, year of disease onset, serogroup, serotype and multilocus variable-number tandem-repeat (VNTR) analysis (MLVA) results are given for each *V*. *cholerae* isolate. Regional three-letter codes: ASH, Ashanti region; CEN, Central region; GAR, Greater Accra region; VOL, Volta region. District three-letter codes: AAN, Asante Akim North; ACC, Accra; ADE, Adentan; AGW, Agona-Swedru; AWS, Awutu-Senya; ASA, Ashaiman; GAE, Ga East; GAS, Ga South; GAW, Ga West; GOE, Gomoa East; GOW, Gomoa West; HOH, Hohoe; HOV, Ho; KPK, Kpone-Katamanso; LEK, Ledzekuku-Krowor; TEM, Tema.

Out of these 92 isolates, 11 isolates from 2011, eight isolates from 2012 and 26 isolates from 2014 were genotyped by MLST, PFGE and MLVA (S1 Fig). All *V*. *cholerae* O1 belonged to the sequence type (ST) 69, while the three non-O1/non-O139 isolates presented with new alleles (isolate 059: *metE* allele 108; isolate 160: *metE* allele 109; and isolate 169: *metE* allele 110 and *pyrC* allele 85) and could not be allocated to any known ST. Theses strains were assigned new ST numbers (isolate 059: ST211, isolate 160: ST212 and isolate 169: ST213; corresponding to IDs 196, 197 and 198 in the *V*. *cholerae* pubmlst database: http://pubmlst.org/vcholerae/), which are closest related to the already known ST40 and ST39. As shown in Fig 3, using a cutoff of 95%, PFGE divided all strains into three main clusters and 11 pulsotypes (Fig 3). All 2011 isolates clustered together (cluster A) as 4 different pulsotypes. Isolates from 2012 belonged to 4 different pulsotypes dispersed in clusters A, B and C. All *V*. *cholerae O1* from 2014 showed the same pulsotype and were grouped in cluster B. Finally, the non-O1/non-O139 isolates collected in 2014 showed different pulsotypes and were grouped outside clusters A, B or C. Neither regional nor district specific pulsotypes had been detected.

**Fig 4:**
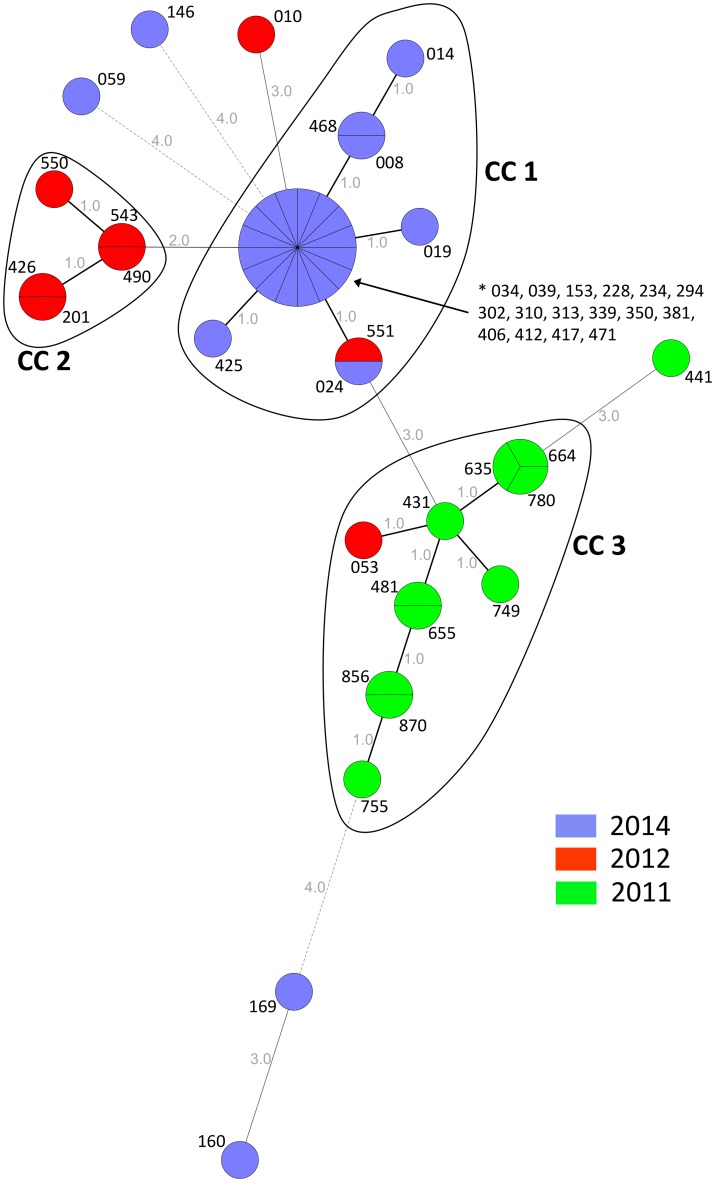
Minimum spanning tree of multilocus variable-number tandem-repeat (VNTR) analysis (MLVA) for *Vibrio cholerae* isolates (n = 45) by year of disease onset. Clonal complexes (CC 1, CC 2, CC 3) were defined as isolates connected through a chain of single-locus variants. Grey figures indicate the number of different alleles. Three-digit codes present the laboratory isolate number.

MLVA differentiated the isolates into three clonal complexes (CC1-CC3) and 22 genotypes (Fig 4). The majority of 22 out of 26 isolates from 2014 clustered within CC1 together with one isolate from 2012. Distinct from the CC1 were the three non-O1/non-O139 and one O1 *V*. *cholerae* isolate (Isolate 146) from Ledzekuku-Krowor District (Greater Accra Region). For 2012, five out of eight isolates grouped together in CC2, while the other three isolates were genetically distinct, within or close to CC1 and CC3. Similarly, 10 of the 11 isolates from 2011 were classified into CC3, and one strain clustered outside with three alleles difference to the other CC3 strains. Analogous to the PFGE distribution, subtypes did not cluster by region or by district.

## Discussion

This study investigated the largest cholera outbreak in Ghana in more than a decade caused by *Vibrio cholerae* O1 biotype El Tor carrying the classical cholera toxin. The current seventh pandemic is caused by El Tor biotype, which almost completely replaced the classical biotype of previous pandemics [20]. A recent sweep originated from Asia, in which the classical cholera toxin gene selectively replaced the El Tor toxin gene, as currently observed in the Ghanaian outbreak [21,22]. It has been suggested that the recent emergence of a classical *ctx*B variant in the predominant global El Tor linage is associated with increased clinical virulence, linked with a possible increase in cholera toxin production [23].

Despite the high number of cases, the fatality rate remained below the WHO target of 1% during the 2014 outbreak [1], in contrast to reported numbers from other major cholera outbreaks on the African continent, such as from Kenya (CFR = 2.3%) or Zimbabwe (CFR = 4.7%) [9,24]. In line with the Ghanaian data, older age is a risk factor for mortality as reported from other outbreaks [25]. However, case fatality data from hospital based mortality figures have to be interpreted with caution, because in resource limited settings, many deaths may occur before patients are able to reach the hospital and might have escaped notification/reporting [4].

Ghana has been classified as a cholera endemic country based on epidemiological data, but this has not yet been confirmed using molecular genotyping methods [4]. While surveillance system data suggest a spread from a single source within the capital Accra to surrounding regions, molecular subtyping data hints to the existence of co-circulating *V*. *cholerae* strains distinct from the major outbreak strain.

Previous outbreak investigations have demonstrated the need to distinguish between clonal and multi-clonal outbreaks in order to focus prevention measures [11]. As shown in previous studies MLVA proves to be more discriminatory than PFGE to distinguish *V*. *cholerae* O1 strains [17,26–28]. Using this technique, both multi-clonal and clonal outbreaks have been reported from Africa. An outbreak in Guinea in 2012 was ascribed to a single *V*. *cholerae* clone, probably imported by fishermen from Sierra Leone. The closest known relative was a strain isolated in Bangladesh [29]. In contrast, a genotyping study in Kenya traced the 2009/2010 cholera outbreak to multiple genetic lineages [9]. The present study suggests annual dominant MLVA types, which circulated in Ghana during the years 2011, 2012 and 2014, with minor MLVA types occurring during the same outbreak period. The detection of different distinct genotypes among annual outbreak strain collections between 1970 and 2006 in Ghana and Nigeria supports these findings [14,30]. Outside of Africa, similar co-circulating genotypes have been observed in Thailand between 2007 and 2010 [31]. These data suggest that in Ghana *V*. *cholerae* has perhaps an endemic reservoir in the environment and selection pressure results in a highly heterogeneous population of *V*. *cholerae* with a few strains evolving into pathogenic clones [32,33], however the present study is not able to proof this hypothesis and a recurrent annual introduction of strains, although unlikely, cannot be ruled out.

The onset of the 2014 outbreak in Ghana coincided with the start of the rainy season in May. Environmental studies conducted in Haiti have demonstrated a seasonal correlation between *Vibrio* onset and increases in precipitation and water temperature [34]. This is consistent with observations from countries with endemic *V*. *cholerae* O1, where the seasonal rise in water temperatures or the onset of the rainy season serve as triggers for the proliferation of *V*. *cholerae* O1 in the environment, preceding seasonal cholera epidemics [34]. It has been shown that long-term survival of a particular genotype may be attained in watery environments or in humans with no signs of cholera [35]. *V*. *cholerae* infections might occur when people come into close contact with such aquatic reservoirs, particularly during rainy seasons, natural disasters, such as floods or cyclones, but also during droughts, when water sources are over-exploited and polluted [11]. Consequently, cholera outbreaks may be triggered predominantly in large densely populated urban areas, as shown for Abidjan, Cotonou, Douala or Lomé [36–39].

However, no perennial environmental reservoir of toxigenic *V*. *cholerae* O1 has yet been identified in West Africa, which could be attributed to the lack of appropriate studies [11]. With environmental cholera sources being suspected in Ghana, enhanced monitoring of aquatic reservoirs and drinking water should be taken into consideration, particularly within urban agglomerations. People involved in any kind of water related activities, such as fishing, may be targeted by prevention measures.

This study identified three non-O1/non-O139 *V*. *cholerae* strains, which are naturally present in aquatic ecosystems, often non-pathogenic or associated with only mild disease [23]. However, depending on virulence factors they may cause severe diarrhoea, similar to pandemic *V*. *cholerae* O1 strains [23]. In this study none of those strains carried the cholera toxin. Due to the non-specific case definition it remains unclear whether these strains caused the watery diarrhea or those patients were co-infected with other enteric pathogens.

Of serious concern is the increase of antimicrobial resistances between 2011 and 2014, as it has been shown that effective antibiotics shorten the duration of diarrhoea, reduce the volume of stool losses by up to 50% and reduce the duration of shedding of viable organisms in stool from several days to 1–2 days [40]. Multidrug resistance (MDR), defined as resistance to at least three classes of antimicrobial agents normally effective against *V*. *cholerae*, was detected in 95% of 2014 isolates, while no MDR isolates were present in 2011. In a study on Ghanaian isolates from 2006 31% of *V*. *cholerae* were reported to be MDR [30]. Resistance to ampicillin, ciprofloxacin, nalidix acid, and sulfamethoxazole/trimethoprim have all been described before from various countries worldwide, including Ghana and other regions of the African continent [24,30,41–44]. An increase in the minimal inhibitory concentrations to quinolones has been noticed since the 1980s and has become common in endemic areas being associated with treatment failures [40]. Therefore, careful and regular laboratory monitoring of the antibiotic sensitivity of circulating environmental and outbreak strains is recommended in all settings. Isolates within this study were not screened for macrolides resistance and Extended-spectrum β-lactamases (ESBL). However, ESBL producing *V*. *cholerae* strains are rarely reported. Nevertheless, they have been detected sporadically in Ghana in 2006 and South Africa in 2009 [24,30].

This study has some limitations. The surveillance database is based on a non-specific case definition. Only about half of all specimens tested in peripheral hospitals were positive for *V*. *cholerae*. Whether this is due to the case definition itself or rather due to sampling, transport or storage conditions remains uncertain. However, WHO estimates that the official reported cholera cases represent 5–10% of the actual disease burden [4]. The database is restricted to six Southern Ghanaian regions around the outbreak epicentre (Greater Accra, Central, Western, Eastern, Ashanti, Volta) for which data quality was adequate for the analysis. The *V*. *cholerae* isolates originate from the same Southern regions. Therefore, results cannot be generalized to the whole of Ghana and outbreak numbers differ from official WHO numbers.

This study uses well-established subtyping methods, such as MLST, PFGE and MLVA. Future studies should consider Whole Genome Sequencing approaches in order to place West African *V*.*cholerae* isolates into the regional and global phylogenetic framework.

### Conclusion

The Ghanaian Cholera outbreak in 2014 was caused by *Vibrio cholerae* O1 biotype El Tor carrying the classical cholera toxin. Molecular subtyping data of three outbreak years illustrate major annual outbreak clusters with co-circulating genetically distant genotypes, which might hint to an endemic reservoir of *V*. *cholerae* in Ghana. Public health authorities must be vigilant and take steps to prevent cholera transmission through aquatic reservoirs, particularly within urban agglomerations during the start of the rainy season. Considering the rapidly emerging multidrug resistance among *V*. *cholerae* isolates, laboratories are encouraged to monitor antimicrobial susceptibility closely.

## Supporting Information

S1 Fig Geographical distribution of *Vibrio cholerae* isolates selected for further molecular characterization. From all isolates identified within a two-week period in a specific district, one isolate was randomly selected for multilocus sequence typing (MLST), Pulse-field gel electrophoresis (PFGE) and multilocus variable-number tandem-repeat (VNTR) analysis (MLVA), resulting in a subset of 45 isolates. The figure was produced with Arc GIS 10.0 (ESRI: ArcGis Desktop: Release 10.2011).
(TIF)

S1 Dataset Complete dataset for the laboratory analysis. (TXT)

S1 Checklist STROBE Checklist. (DOCX)
