# Supplementary figures and images for: Molecular Epidemiology and Antibiotic Susceptibility of Vibrio cholerae Associated with a Large Cholera Outbreak in Ghana in 2014
Source: PLoS Negl Trop Dis. 2016 May 27;10(5):e0004751. doi: 10.1371/journal.pntd.0004751 (PMC4883745; doi:10.1371/journal.pntd.0004751)

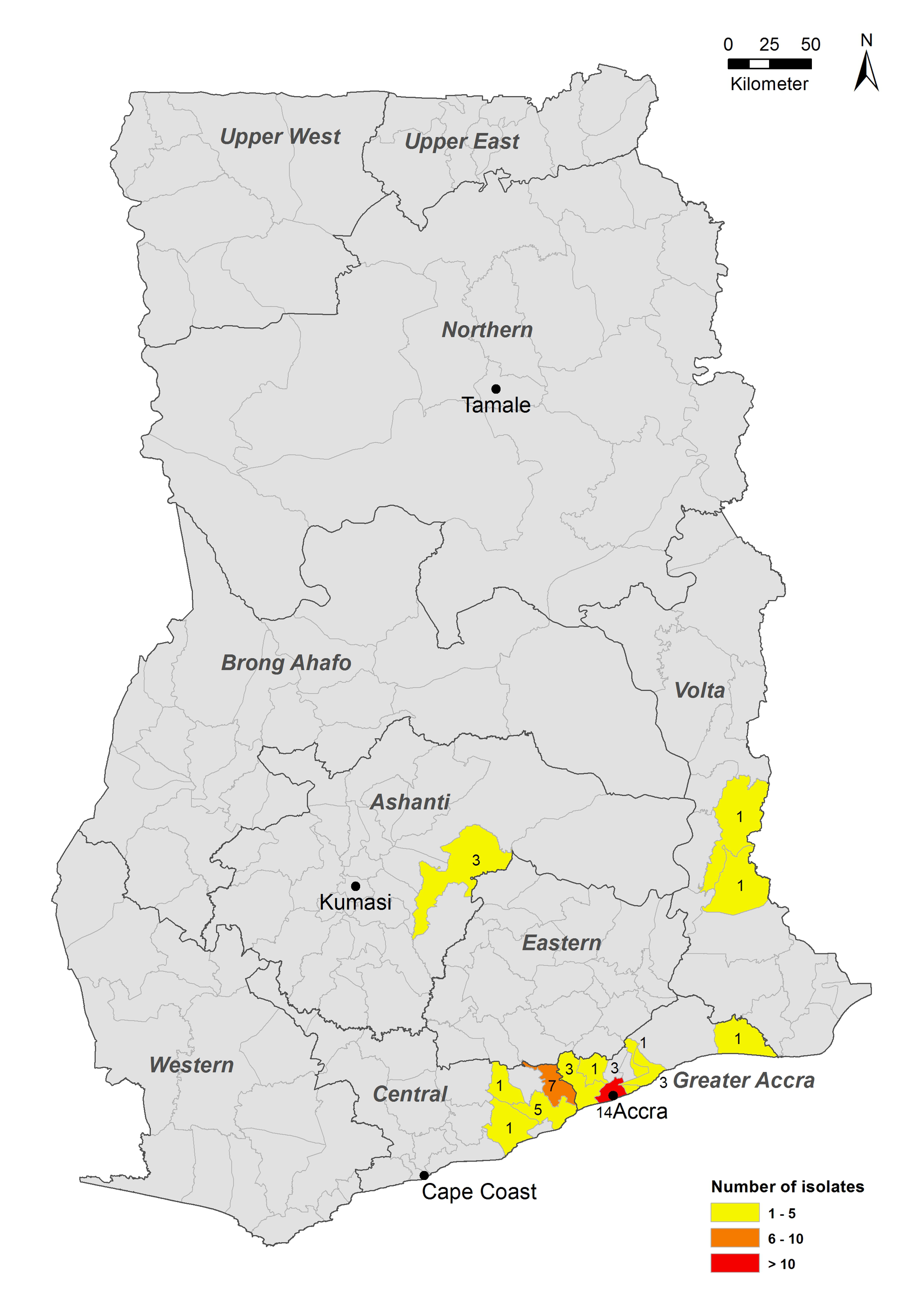

Supplement: S1 Fig — From all isolates identified within a two-week period in a specific district, one isolate was randomly selected for multilocus sequence typing (MLST), Pulse-field gel electrophoresis (PFGE) and multilocus variable-number tandem-repeat (VNTR) analysis (MLVA), resulting in a subset of 45 isolates. The figure was produced with Arc GIS 10.0 (ESRI: ArcGis Desktop: Release 10.2011). (TIF) [file pntd.0004751.s001.tif]
