# Supplementary material for: Molecular Epidemiology and Antibiotic Susceptibility of Vibrio cholerae Associated with a Large Cholera Outbreak in Ghana in 2014
Source: PLoS Negl Trop Dis. 2016 May 27;10(5):e0004751. doi: 10.1371/journal.pntd.0004751 (PMC4883745; doi:10.1371/journal.pntd.0004751)
Supplement: S1 Checklist — (DOCX) [file pntd.0004751.s003.docx]

STROBE Statement—checklist of items that should be included in reports of observational studies

|  | | Item No. | | Recommendation | Line  No. | Relevant text from manuscript | |
| --- | --- | --- | --- | --- | --- | --- | --- |
| **Title and abstract** | | 1 | | (*a*) Indicate the study’s design with a commonly used term in the title or the abstract | 1 | Titel: Molecular Epidemiology … | |
|  |  |  |  | (*b*) Provide in the abstract an informative and balanced summary of what was done and what was found | 33 - 43 | Abstract: See methodology principal findings | |
| Introduction | | | | | |  | |
| Background/rationale | | 2 | | Explain the scientific background and rationale for the investigation being reported | 83 | Causes of this sudden increase in case numbers might be very diverse and calls for investigation in a timely manner so as to implement preventive measures accordingly | |
| Objectives | | 3 | | State specific objectives, including any prespecified hypotheses | 93 | This study aims to describe the 2014 cholera epidemic in Ghana and uses molecular subtyping techniques to detect responsible newly emerging or multi-clonal strains, which are then compared to strains that circulated during the 2011 and 2012 epidemics. Results will advise public health authorities whether to focus on monitoring of endemic environmental reservoirs or rather on surveillance of mobile populations, with cross-border epidemiological collaborations to prevent importation of *V. cholerae*. | |
| Methods | | | | | |  | |
| Study design | | 4 | | Present key elements of study design early in the paper | 102 | Within the Ghana Health Service, the Disease Surveillance Service supported by the National Public Health & Reference Laboratory (NPHRL) conducts cholera surveillance in Ghana | |
| Setting | | 5 | | Describe the setting, locations, and relevant dates, including periods of recruitment, exposure, follow-up, and data collection | 116  130 | …all suspected cholera cases in the surveillance database from the year 2014 were extracted…  …from all *V. cholerae* isolates collected in 2011, 2012 and 2014, … at the NPHRL, 92 were randomly selected… | |
| Participants | | 6 | | (*a*) *Cohort study*—Give the eligibility criteria, and the sources and methods of selection of participants. Describe methods of follow-up  *Case-control study*—Give the eligibility criteria, and the sources and methods of case ascertainment and control selection. Give the rationale for the choice of cases and controls  *Cross-sectional study*—Give the eligibility criteria, and the sources and methods of selection of participants | 104  160 | …defined according to the WHO standard case definition: If cholera is not known to be present in the area, a case of cholera is considered in a patient ≥5 years with severe dehydration or death from acute watery diarrhea, while during a cholera epidemic every patient aged ≥5 years with acute watery diarrhea and/or vomiting is considered as a case. …  From all isolates identified within a two week period in a specific district, one isolate was randomly selected for further molecular characterization, resulting in a subset of 45 isolates. | |
|  |  |  |  | (*b*) *Cohort study*—For matched studies, give matching criteria and number of exposed and unexposed  *Case-control study*—For matched studies, give matching criteria and the number of controls per case | NA |  | |
| Variables | | 7 | | Clearly define all outcomes, exposures, predictors, potential confounders, and effect modifiers. Give diagnostic criteria, if applicable | NA |  | |
| Data sources/ measurement | | 8* | | For each variable of interest, give sources of data and details of methods of assessment (measurement). Describe comparability of assessment methods if there is more than one group | 109 | Standardized line lists with suspected cholera cases are provided on a weekly basis by the District Health Management Teams to the Central Disease Surveillance Service in Accra, which collates information on name, place of residence, sex, age, disease onset, disease outcome and hospitalization of cases. | |
| Bias | | 9 | | Describe any efforts to address potential sources of bias | NA |  | |
| Study size | | 10 | | Explain how the study size was arrived at |  | This is a molecular epidemiology study, in which no associations between exposures and outcomes are calculated. No samples size calculation was performed. | |
| Quantitative variables | 11 | | Explain how quantitative variables were handled in the analyses. If applicable, describe which groupings were chosen and why | | 117  124 | Continuous variables were summarized as means with standard deviation (median with interquartile range for non-normally distributed variables) and dichotomous or categorical variables were summarized as proportions/percentages.  For the spatio-temporal visualisation, case data/district were then aggregated in six temporal groups, each covering five outbreak weeks. | |
| Statistical methods | 12 | | (*a*) Describe all statistical methods, including those used to control for confounding | |  | No statistical methods have been used | |
|  |  |  | (*b*) Describe any methods used to examine subgroups and interactions | |  | No statistical subgroup analysis or interactions have been performed | |
|  |  |  | (*c*) Explain how missing data were addressed | | 120 | Missing values were excluded from the analysis, thus the denominators for some comparisons differ. | |
|  |  |  | (*d*) *Cohort study*—If applicable, explain how loss to follow-up was addressed  *Case-control study*—If applicable, explain how matching of cases and controls was addressed  *Cross-sectional study*—If applicable, describe analytical methods taking account of sampling strategy | | NA |  | |
|  |  |  | (*e*) Describe any sensitivity analyses | | NA |  | |
| Results | | | | | | |  |
| Participants | 13* | | (a) Report numbers of individuals at each stage of study—eg numbers potentially eligible, examined for eligibility, confirmed eligible, included in the study, completing follow-up, and analysed | | 185  190  203  235  239 | …20,185 cases of cholera were reported to the Ghanaian Disease Surveillance Center in 2014. The date of disease onset was reported for 20,120 cases…  …Age was reported for 19,863 cases…  …Laboratory testing was performed in regional laboratories for 496 out of 20,120…  … Biotyping and serotyping of 92 *V. cholerae* isolates…  …Out of these 92 isolates, 11 isolates from 2011, eight isolates from 2012 and 26 isolates from 2014 were genotyped… | |
|  |  |  | (b) Give reasons for non-participation at each stage | | 185  203 | The date of disease onset was reported for 20,120 cases…  …Laboratory testing was performed in regional laboratories for 496 out of 20,120… | |
|  |  |  | (c) Consider use of a flow diagram | | NA |  | |
| Descriptive data | 14* | | (a) Give characteristics of study participants (eg demographic, clinical, social) and information on exposures and potential confounders | | 186-196 | First section of the result part + figure 1 | |
|  |  |  | (b) Indicate number of participants with missing data for each variable of interest | | 185  190  203  235  239 | …20,185 cases of cholera were reported to the Ghanaian Disease Surveillance Center in 2014. The date of disease onset was reported for 20,120 cases…  …Age was reported for 19,863 cases…  …Laboratory testing was performed in regional laboratories for 496 out of 20,120…  … Biotyping and serotyping of 92 *V. cholerae* isolates…  …Out of these 92 isolates, 11 isolates from 2011, eight isolates from 2012 and 26 isolates from 2014 were genotyped… | |
|  |  |  | (c) *Cohort study*—Summarise follow-up time (eg, average and total amount) | | NA |  | |
| Outcome data | 15* | | *Cohort study*—Report numbers of outcome events or summary measures over time | | NA |  | |
|  |  |  | *Case-control study—*Report numbers in each exposure category, or summary measures of exposure | | NA |  | |
|  |  |  | *Cross-sectional study—*Report numbers of outcome events or summary measures | | 186 - 210 | Figure 1, Detiails are given in the section “Epidemiology of the 2014 outbreak” | |
| Main results | 16 | | (*a*) Give unadjusted estimates and, if applicable, confounder-adjusted estimates and their precision (eg, 95% confidence interval). Make clear which confounders were adjusted for and why they were included | | 218 - 281 | No associations were calculated. The main results are presented between lines 218 and 281, characterizing the subtypes of *V. cholerae* strains | |
|  |  |  | (*b*) Report category boundaries when continuous variables were categorized | | NA |  | |
|  |  |  | (*c*) If relevant, consider translating estimates of relative risk into absolute risk for a meaningful time period | | NA |  | |

| Other analyses | 17 | Report other analyses done—eg analyses of subgroups and interactions, and sensitivity analyses | 218-281 | The molecular characterization of the *V. cholerae* isolates was presented in the last part of the result section. 218-281 |
| --- | --- | --- | --- | --- |
| Discussion | | | | |
| Key results | 18 | Summarise key results with reference to study objectives | 284  308 | …Ghana in more than a decade caused by *Vibrio cholerae* O1 biotype El Tor carrying the classical cholera toxin…  …molecular subtyping data hints to the existence of co-circulating *V. cholerae* strains distinct from the major outbreak strain… |
| Limitations | 19 | Discuss limitations of the study, taking into account sources of potential bias or imprecision. Discuss both direction and magnitude of any potential bias | 297  299 | …have to be interpreted with caution…  Another limitation of the database… |
| Interpretation | 20 | Give a cautious overall interpretation of results considering objectives, limitations, multiplicity of analyses, results from similar studies, and other relevant evidence | 284-371 | The molecular subtyping results and clinical symptoms are discussed with reference to the literature. |
| Generalisability | 21 | Discuss the generalisability (external validity) of the study results | 329  341 | This is consistent with observations from countries with endemic *V. cholerae* O1  However, no perennial environmental reservoir of toxigenic *V. cholerae* O1 has yet been identified in West Africa, which could be attributed to the lack of appropriate studies |
| Other information | |  | | |
| Funding | 22 | Give the source of funding and the role of the funders for the present study and, if applicable, for the original study on which the present article is based |  | Funding statement submitted |

*Give information separately for cases and controls in case-control studies and, if applicable, for exposed and unexposed groups in cohort and cross-sectional studies.
